# Soft Compression for Lossless Image Coding Based on Shape Recognition

**DOI:** 10.3390/e23121680

**Published:** 2021-12-14

**Authors:** Gangtao Xin, Pingyi Fan

**Affiliations:** 1 Department of Electronic Engineering, Tsinghua University, Beijing 100084, China; xgt19@mails.tsinghua.edu.cn; 2 Beijing National Research Center for Information Science and Technology (BNRist), Beijing 100084, China

**Keywords:** lossless image compression, information theory, statistical distributions, compressible indicator function, image set compression

## Abstract

Soft compression is a lossless image compression method that is committed to eliminating coding redundancy and spatial redundancy simultaneously. To do so, it adopts shapes to encode an image. In this paper, we propose a compressible indicator function with regard to images, which gives a threshold of the average number of bits required to represent a location and can be used for illustrating the working principle. We investigate and analyze soft compression for binary image, gray image and multi-component image with specific algorithms and compressible indicator value. In terms of compression ratio, the soft compression algorithm outperforms the popular classical standards PNG and JPEG2000 in lossless image compression. It is expected that the bandwidth and storage space needed when transmitting and storing the same kind of images (such as medical images) can be greatly reduced with applying soft compression.

## 1. Introduction

Image compression is to reduce the required number of bits as much as possible when representing an image. In this process, the fidelity of the reconstructed image and original image should be higher than the reference value. Image compression often consists of two parts, encoding and decoding. Encoding is to convert the input image into a binary code stream with a coding method, while decoding, the reverse process of encoding, aims to restore the original image from the binary code stream.

There are two categories of image compression: lossy compression and lossless compression. Lossy compression allows the reconstructed image to be different from the original image, but it is still visually similar. However, lossless compression requires the reconstructed image to be exactly the same as the original image, which leads to the compression ratio being much smaller than that of lossy compression. Although the compression ratio of lossy compression is higher, lossless compression is significant in many fields. Lossless compression should be applied when errors cannot be tolerated or the image has significant value, such as medical images, precious cultural relics, deep space exploration, deep sea exploration and digital libraries.

Supposing that we regard an image as a random process, the minimum expected codeword length per pixel $L_{n}^{*}$ will approach the entropy rate asymptotically, as shown in Formula (1). However, for an actual image, the upper and lower bounds of Formula (1) cannot be theoretically calculated because one cannot know the spatial correlation of pixels clearly.

(1) $$\frac{H(X_{1},X_{2},\ldots ,X_{n})}{n}\leq L_{n}^{*}<\frac{H(X_{1},X_{2},\ldots ,X_{n})}{n}+\frac{1}{n}$$

where $H(X_{1},X_{2},\ldots ,X_{n})$ is the joint entropy of the symbol series $\left\{X_{i},i=1,2,\ldots ,n\right\}$.

It is impossible to reach the entropy rate for an image. All we can do is to make great efforts to get close to it. Xin et al. [1] proposed soft compression based on information theory and statistical distribution. It uses shapes and locations to reflect the spatial correlation of an image, trying to achieve better compression performance.

In the literature, most of the image compression methods mainly consider three aspects to reduce the required number of bits when representing an image: coding redundancy, spatial redundancy and irrelevant information. Coding redundancy refers to the diverse probability of each pixel value in an image so that the average length can be reduced from the perspective of coding. Spatial redundancy means that pixels are spatially related. The repeated information can be omitted because the pixel is similar to or depends on adjacent pixels [2]. Irrelevant information refers to an image containing information irrelevant to the human visual system or purpose, which leads to redundancy. Image compression techniques usually improve the algorithm performance from one or several aspects.

### 1.1. Image Compression Method

Huffman coding [3] is an extraordinary method to eliminate coding redundancy for a stream of data. Arithmetic coding [4] and Golomb coding [5] are also approaches to eliminating coding redundancy. They all require accurate probability models of input symbols. Run-length coding [6] represents runs of identical intensities by a new coding value and length, but it may result in data expansion when there are few runs of identical intensities [2]. LZW coding [7] is a method to remove spatial redundancy, assigning fixed-length codewords to variable length sequences of source symbols, but it is easy to cause data explosion, especially when the input is of a large size or irregular.

Image predictive coding is a means of transforming spatial redundancy into coding redundancy through prediction error, which is an entry point of image compression. The paper [8] applies prediction to discrete wavelet transform subbands. In [9], it predicts the probability of a high-resolution image, conditioned on the low-resolution input, and uses entropy coding to compress the super-resolution operator. The Consultative Committee for Space Data Systems (CCSDS) [10] is a multi-national forum for the development of communications and data systems standards for spaceflight, which has proposed several excellent image lossless compression algorithms [11,12].

Transform coding [13,14,15] maps an image from the spatial domain to transform domain, and then encodes the coefficients of the transform domain to achieve the compression effect. It reduces the irrelevant information in an image from the visual point of view. As a tool of multi-resolution analysis, wavelet coding [16,17,18] has been widely concerned and applied. In [19], it uses both wavelet and fractional transforms for lossless image compression. In [20], it designs a reversible integer-to integer wavelet filter to achieve the effect of lossless compression. In [21], it describes edge-based and prediction-based transformations for image compression.

With the development of neural networks, image compression methods based on learning have received a lot of attention [15,22,23,24]. Recent works are mainly in the area of lossy compression, which are based on convolutional neural networks (CNNs) [25,26,27,28,29], recurrent neural networks (RNNs) [30], generative adversarial networks (GANs) [31] and the context model  [32,33,34]. Learning-based lossless image compression methods [35,36,37,38,39] use neural networks instead of the traditional encoder and decoder to achieve image compression. PixelCNN [40] and PixelCNN++ [41] as well as the methods based on bits-back coding [42,43] and flow models [44,45] shorten the distance between information theory and machine learning. In [46], it proposes a fully parallel hierarchical probabilistic model with auxiliary feature representations. The neural network of long short-term memory (LSTM) can also be used to build a predictor for lossless image compression [47].

As for image coding standards, there are some mature instances (PNG [48], JPEG XR [49], JPEG-LS [50], and WebP [51]). JPEG [52] and JPEG2000 [53] are based on discrete cosine transform [54] and wavelet transform [55], respectively. FLIF [56] is based on meta-adaptive near-zero integer arithmetic coding.

### 1.2. Related Work

Soft compression has two special properties. (1) It uses shapes and corresponding locations to represent an image. (2) Its codebook is generated through data-driven means. The earliest coding approaches with symbols and locations can be traced back to symbol-based coding [57]. A picture is denoted as a set of frequently occurring sub-images, called symbols. Storing repeated symbols only once can compress images significantly, especially in document storage, where the symbols are usually character bitmaps that are repeated many times. However, symbol-based coding is hard to generalize to other scenarios, owing to the need of redesigning symbols. Some methods are also based on shape coding [58,59], but none of them consider both shapes and locations at the same time. Fractal block coding [60] relies on the assumption that image redundancy can be efficiently exploited through self-transformability on a blockwise basis and it can approximate an original image by a fractal image. However, it is mainly used in lossy compression because it is tough to find a great deal of identical blocks from only one image.

Finding similar features from a database has been an active research topic in the field of image compression in recent years. In [61], an off-the-shelf image database is used to find patches that are visually similar to each region of interest of the unknown input image, according to associated local descriptors. These patches are then warped into the input image domain according to interest region geometry and seamlessly stitched together. In [62], the authors make use of external image contents to reduce visual redundancy among images with SIFT descriptors [63]. In [64], a method is proposed for cloud-based image coding that no longer compresses images pixel by pixel and instead tries to describe images and reconstruct them from a large-scale image database via the descriptions. In [65], the authors adopt a semi-local approach to exploit inter-image correlation by using information from external image similarities. In [66], a cloud storage system is proposed that reduces the storage cost of all uploaded JPEG photos at the expense of a controlled increase in computation mainly during the download of a requested image subset. In [67], it proposes a novel framework for image set compression based on the rate-distortion optimized sparse coding.

### 1.3. Soft Compression

Soft compression was first proposed in [1], using shapes and locations to represent a binary image. The set of shapes used in soft compression is not designed by experts, but searched from a dataset. Different datasets may have diverse codebooks, which ensures the adaptability of soft compression. Moreover, the codebook corresponding to each dataset is complete, containing all the possibilities of the smallest shape, which makes soft compression workable. Due to the adaptability and completeness of codebooks of soft compression, they can always achieve lossless compression for any image and any codebook. When the codebook and image match well, it will result in a better compression ratio.

The main idea of soft compression is to represent an image with a set of triplets $\left(x_{i},y_{i},S_{i}\right)$, where $\left(x_{i},y_{i}\right)$ denotes the position of shape $S_{i}$ in an image. The set of shapes is obtained by searching in the training set. After that, the set of codewords and the codebook can be obtained by variable length coding for the set of shapes according to the size and frequency of each shape. When an image is encoded, it is transformed into a set of triplets $\left(x_{i},y_{i},C_{i}\right)$ according to the codebook, where $C_{i}$ is the codeword of shape $S_{i}$. On the other hand, we also require to decode the compressed data into a set of triplets $\left(x_{i},y_{i},S_{i}\right)$ according to the codebook when decoding. Finally, we fill shapes in the corresponding locations to reconstruct the original image.

Soft compression is instrumental in reducing storage space and communication bandwidth in the process of transmitting and storing the same kind of images. When two sides communicate, the transmitter only needs to send the compressed data instead of the whole picture to the receiver in the case that both sides have identical codebooks.

In this paper, we try to answer the following two fundamental problems for lossless image compression and design a novel image coding algorithm based on soft compression, outperforming the popular classical standards, PNG and JPEG2000.

① How do we detect an image to be compressible in theory? In other words, what is the value of the compressible indicator function for an image?

② If one image is compressible, how do we find a way to compress it through increasing the value of the compressible indicator function?

This paper is organized as follows. We first introduce a new concept, a compressible indicator function with regard to images based on information theory. Then, we use it to evaluate the performance of soft compression in Section 2. In Section 3, some soft compression algorithms for binary image, gray image and multi-component image are proposed. Then, we give the experimental results and theoretical analysis in Section 4. Finally, we conclude this paper in Section 5.

## 2. Theory

Digital images have coding and spatial redundancy, which makes compression feasible. Soft compression is committed to eliminating these two kinds of redundancy simultaneously by filling an image with shapes. In this section, we introduce the theory of soft compression.

### 2.1. Preliminary

#### 2.1.1. Information Theory

Information theory provides the answer to the lower bound of data compression. For an image, the minimum number of bits required per pixel is given by Formula (1), which is the entropy rate of a random process.

**Definition** **1.** *Let Z be a discrete random variable with alphabet Z and probability mass function p(z)=Pr{Z=z},z∈Z. The entropy [68] H(Z) of a discrete random variable Z is defined as*

(2) $$H\left(Z\right)=-\sum_{z\in Z}p\left(z\right)\log p\left(z\right)$$

Entropy is a measure of the average uncertainty of a random variable. Moreover, it points out the minimum cost of encoding the random variable [69]. In this paper, we take all logarithms to base 2 so that entropy is measured in bits unless otherwise specified.

**Definition** **2.** *Suppose that Z is a random variable with only two events, i.e.,*

(3) $$Z=\left\{\begin{array}{c} 0\;\;\;with\;probability\;p\\ 1\;\;\;with\;probability\;1-p \end{array}\right.$$

*Then the entropy of Z is given by*

(4) $$H(Z)=-p\log p-(1-p)\log(1-p)\overset{\mathrm{def}}{\;=}H(p)$$

Note that H(p) is a concave function of *p* and equals 1 when p=0.5. In the case of p=0 or p=1, H(p) reaches its minimum value of 0. Moreover, the random variable becomes a constant due to the lack of randomness.

If each pixel in an image is independently and identically distributed, the minimum expected number of bits required is the entropy. However, for an actual image, the probability distribution of each pixel cannot be independently and identically distributed. Due to the spatial correlation, the minimum expected number of bits required for a pixel is the entropy rate of the random process corresponding to an image. How to evaluate it is still an open problem in the literature.

#### 2.1.2. Image Fundamentals

Let *I* denote a digital image with intensity levels in the range [0,D−1] whose row and column dimensions are *M* and *N*, respectively. $r_{k}$ is the *k*-th intensity value. $n_{k}$ is the number of pixels with intensity $r_{k}$ in the image *I* [2]. We define *X* as a discrete random variable with probability mass function $p(x_{k})$

(5) $$p\left(x_{k}\right)=\mathrm{Pr}\left\{X=r_{k}\right\}=\frac{n_{k}}{MN}\;\;k=0,1,2,\ldots ,D-1$$

*X* reflects the frequency distribution of pixel intensity values in an image.

We define *Y* as the same random variable as *X*, but *Y* removes event $r_{0}$. Let $p=\mathrm{Pr}\{X=r_{0}\}$, then

(6) $$p\left(y_{k}\right)=\frac{p(x_{k})}{1-p}\;\;\;k=1,2,\ldots ,D-1$$

*Y* indicates the frequency distribution of remaining intensity values with removing $r_{0}$ from *X*. When p=1, *X* will change from a random variable to a constant. For this reason, we mainly consider the case where p<1.

**Lemma** **1.** *Let H(X) and H(Y) denote the entropy of X and Y, respectively. Then,*

(7) $$H\left(Y\right)=\frac{H(X)-H(p)}{1-p}$$

*where* H(p) *comes from Definition 2.*

**Proof.**  (8) $$\begin{array}{cc} H(Y) & =-\sum_{k=1}^{D-1}p\left(y_{k}\right)\log p\left(y_{k}\right) \end{array}$$

(9) $$\begin{array}{cc} \; & =-\sum_{k=1}^{D-1}p\left(y_{k}\right)\log p(x_{k})+\sum_{k=1}^{D-1}p\left(y_{k}\right)\log\left(1-p\right) \end{array}$$

(10) $$\begin{array}{cc} \; & =-\sum_{k=1}^{D-1}\frac{p(x_{k})}{1-p}\log p\left(x_{k}\right)+\log\left(1-p\right) \end{array}$$

(11) $$\begin{array}{cc} \; & =\frac{1}{1-p}\left[-\sum_{k=1}^{D-1}p\left(x_{k}\right)\log p\left(x_{k}\right)\right]+\log\left(1-p\right) \end{array}$$

(12) $$\begin{array}{cc} \; & =\frac{1}{1-p}\left[H\left(X\right)+p\log p\right]+\log\left(1-p\right) \end{array}$$

(13) $$\begin{array}{cc} \; & =\frac{H(X)+p\log p+(1-p)\log(1-p)}{1-p} \end{array}$$

(14) $$\begin{array}{cc} \; & =\frac{H(X)-H(p)}{1-p} \end{array}$$

□

### 2.2. Soft Compression

Soft compression is a lossless image compression method which aims to fill images with shapes and locations. The purpose of soft compression is to find essential and representative shapes.

We define S and C as the finite set of shapes and codewords with soft compression, respectively. Let *T* denote the total number of operations in the process of filling an image *I* [1]. Let $S_{i}\in S,i=1,\ldots ,T$ be the shape used in the *i*-th operation. $C_{i}\in C,i=1,\ldots ,T$ is the codeword of $S_{i}$. The location of shape $S_{i}$ is defined as the pixel coordinate pair $\left(x_{i},y_{i}\right)$. We use $F_{i}\left(S_{i}\right),i=1,\ldots ,T$ to represent filling an image with shape $S_{i}$ at position $\left(x_{i},y_{i}\right)$ in the *i*-th operation.

Then, soft compression can be formulated as the following optimization problem:

(15) $$\begin{array}{cc} \min\; & \sum_{i=1}^{T}\left[l\left(C_{i}\right)+l\left(x_{i},y_{i}\right)\right]\\ \; & s.t.\;I=\sum_{i=1}^{T}F_{i}\left(S_{i}\right) \end{array}$$

where $l(C_{i})$ is the number of bits corresponding to $C_{i}$, and $l(x_{i},y_{i})$ is the length of bits needed to represent $\left(x_{i},y_{i}\right)$. The constraint reflects that the original image *I* can be reconstructed with filling shapes through *T* operations. That is, soft compression is lossless. The core goal of designing soft compression algorithm is to find S and C so as to encode images efficiently and effectively.

**Definition** **3.** *We define the compressible indicator function (CIF) with respect to p as*

(16) $$C\left(p\right)=\underset{k}{\sup}\frac{H(p\left(x_{k}\right))\;}{1-p(x_{k})}\;\;\;\;k=0,1,2,\ldots ,D-1$$

*Without loss of generality, we assume $p=p\left(x_{0}\right)=\underset{k}{\sup}p\left(x_{k}\right)$. Then,*

(17) $$C\left(p\right)=\frac{H(p)}{1-p}\;\;\;\;p\in \left(0,1\right)$$

*Moreover, we define compressible indicator value (CIV) as the value of CIF.*

Compressible indicator function C(p) is derived from Lemma 1, which indicates the sparsity of an image. The larger it is, the more capacity can be compressed. The basic properties of the compressible indicator function can be summarized as follows.

**Theorem** **1.** ① *C(p)>0*

② *C(p) is monotonically increasing.*

**Proof.**  ① 0<p<1 implies that H(p)>0, which can reach the conclusion that C(p)>0.

② The derivative of C(p) with respect to *p* is

(18) $$C^{\prime }\left(p\right)=-\frac{\log p}{{\left(1-p\right)}^{2}}>0$$

and therefore, C(p) is monotonically increasing. □

**Definition** **4.** *We use $B_{nb}$ to denote the number of bits required to encode an image with natural binary code, $B_{hf}$ for Huffman coding. Similarly, $B_{sc}^{n}$ is for soft compression where the size of the shapes ranges from 1 to n, i.e., soft compression with n order. $B_{hf,min}$ and $B_{sc,min}^{n}$ represent the minimum values of $B_{hf}$ and $B_{sc}^{n}$, respectively.*

Let $L_{hf}$ be the average number of bits required to represent each pixel with Huffman coding. $L_{sc}^{1}$ is for soft compression where the size of all shapes is one. Then,

(19) $$\begin{array}{cc} \; & B_{nb}=MN\log D \end{array}$$

(20) $$\begin{array}{cc} \; & B_{hf}=MNL_{hf} \end{array}$$

(21) $$\begin{array}{cc} \; & B_{sc}^{1}=MN\left(1-p\right)\left(L_{sc}^{1}+L_{W}\right) \end{array}$$

(22) $$\begin{array}{cc} \; & B_{hf,min}=MNH\left(X\right) \end{array}$$

(23) $$\begin{array}{cc} \; & B_{sc,min}^{1}=MN\left(1-p\right)\left(H\left(Y\right)+L_{W}\right) \end{array}$$

where $L_{W}$ is the average number of bits required to represent a location with soft compression.

**Theorem** **2.** *If $C\left(p\right)\geq L_{W}$ and H(X)>0, the minimum number of bits needed with 1 order soft compression is less than that with Huffman coding, i.e., $B_{sc,min}^{1}\leq B_{hf,min}$. The relative compression ratio $R^{\prime }$ is*

(24) $$R^{\prime }=1+\left(1-p\right)\frac{C\left(p\right)-L_{W}}{H(X)}$$

**Proof.**  (25) $$\begin{array}{cc} B_{sc,min}^{1} & =MN\left(1-p\right)(H\left(Y\right)+L_{W}) \end{array}$$

(26) $$\begin{array}{cc} \; & =MN\left(1-p\right)\left[\frac{H(X)-H(p)}{1-p}+L_{W}\right] \end{array}$$

(27) $$\begin{array}{cc} \; & =MN[H\left(X\right)-H\left(p\right)+\left(1-p\right)L_{W}] \end{array}$$

(28) $$\begin{array}{cc} \; & =B_{hf,min}+MN\left[\left(1-p\right)L_{W}-H\left(p\right)\right] \end{array}$$

Equation (26) uses Lemma 1.

To obtain the result $B_{sc,min}^{1}\leq B_{hf,min}$, we can reach the conclusion that

(29) $$\frac{H(p)}{1-p}=C\left(p\right)\geq L_{W}$$

From Theorem 1, we know that C(p) increases monotonically in (0,1). Due to the non-negativity of entropy and the trivial case for H(X)=0, we mainly consider the case where H(X)>0, then

(30) $$\begin{array}{cc} R^{\prime } & =\frac{B_{hf,min}}{B_{sc,min}^{1}} \end{array}$$

(31) $$\begin{array}{cc} \; & =\frac{B_{sc,min}^{1}-MN\left[\left(1-p\right)L_{W}-H\left(p\right)\right]}{B_{sc,min}^{1}} \end{array}$$

(32) $$\begin{array}{cc} \; & =1-\frac{MN[\left(1-p\right)L_{W}-H\left(p\right)]}{B_{sc,min}^{1}} \end{array}$$

(33) $$\begin{array}{cc} \; & =1-\frac{MN[\left(1-p\right)L_{W}-H\left(p\right)]}{MNH(X)} \end{array}$$

(34) $$\begin{array}{cc} \; & =1+\frac{H\left(p\right)-\left(1-p\right)L_{W}}{H(X)} \end{array}$$

(35) $$\begin{array}{cc} \; & =1+\left(1-p\right)\frac{C\left(p\right)-L_{W}}{H(X)} \end{array}$$

It completes the proof. □

Theorem 2 provides the threshold relationship between $L_{W}$ and C(p). When $L_{W}$ is less than this threshold, the minimum number of bits needed to represent an image with soft compression is lower than that of Huffman coding. In general, we use the minimum value to approximately replace the actual value needed for compression, which is convenient for theoretical analysis. Theorem 2 indicates that for an image whose $C\left(p\right)\geq L_{W}$, soft compression is better than Huffman coding in terms of the compression ratio. It also points out that the higher the compressible indicator value, the higher the compression ratio.

**Figure 1:**
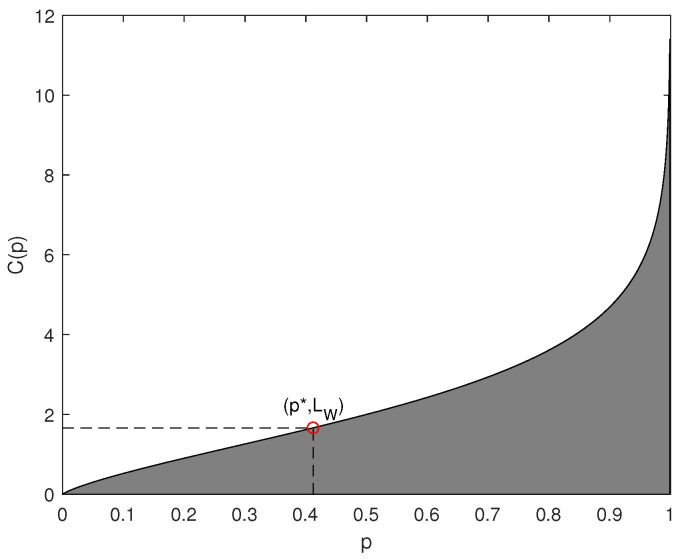
Compressible indicator function versus p.

Figure 1 illustrates the relationship between compressible indicator function C(p) and *p*. Theorem 2 points out the suitability of applying soft compression. Given an image, we should first evaluate the compressible indicator value according to *p*, and then judge whether it is suitable for soft compression. That is to say, if $\left(p,L_{W}\right)$ is in the gray area, the minimum number of bits required with 1 order soft compression is less than that with Huffman coding. As shown in Figure 1, it answers the first fundamental problem of lossless image compression.

**Lemma** **2.** *Let $Y_{n}$ represent the frequency distribution of a shape whose size is n in an image, then*

(36) $$H\left(Y_{n}\right)\leq nH\left(Y\right)$$

**Proof.**  From the independence bound on entropy, one can come to this conclusion. □

**Theorem** **3.** *If $L_{W}$ is a constant for different orders of soft compression, then $B_{sc,min}^{n}\leq B_{sc,min}^{1}$.*

**Proof.**  We use $N_{1}$ to represent the number of shapes with size 1, and $N_{n}$ is the number of shapes with size *n*. The derivation can be seen from (37) to (41). □

(37) $$B_{sc,min}^{n}=N_{1}\left[H\left(Y\right)+L_{W}\right]+N_{2}\left[H\left(Y_{2}\right)+L_{W}\right]+\ldots +N_{n}\left[H\left(Y_{n}\right)+L_{W}\right]$$

(38) $$\;\leq \left[N_{1}+2N2+\ldots +nN_{n}\right]H\left(Y\right)+\left[N_{1}+N_{2}+\ldots +N_{n}\right]L_{W}$$

(39) $$\;\leq \left[N_{1}+2N_{2}+\ldots +nN_{n}\right]\left[H\left(Y\right)+L_{W}\right]$$

(40) $$\;=MN\left(1-p\right)[H\left(Y\right)+L_{W}]$$

(41) $$\;=B_{sc,min}^{1}$$

Theorems 2 and 3 inspire us that in image compression, we can improve the compression ratio from two aspects: one is to increase the compressible indicator value of an image, and the other is to reduce the number of bits required to represent locations. On the one hand, the compressible indicator value can be increased by predictive coding. On the other hand, Golomb coding may be a promising method for encoding the distance between each location and the previous location.

## 3. Implementation Algorithm

In this section, we try to answer the second problem mentioned in Section 1, finding some efficient ways to improve the compression ratio with raising the compressible indicator value. We introduce the soft compression algorithm for different image formats, including binary image, gray image and multi-component image. In fact, three different algorithms vary in specific steps but all try to fill images with shapes and corresponding locations.

### 3.1. Binary Image

The binary image is quite suitable for encoding with soft compression because each pixel has only two intensity values. The probability of $r_{0}$ can always be greater than or equal to 0.5 (through reverse operation), which ensures that the compression indicator value is greater than or equal to 2.

The soft compression algorithm for a binary image was proposed in [1], which had excellent compression performance. Although [1] introduced the algorithm, they did not provide the theoretical analysis. We analyze the experimental results with compressible indicator function in Section 4.

### 3.2. Gray Image

One of the most important steps of the soft compression algorithm for a gray image is to divide the images into two layers, the shape layer and detail layer. In fact, the compressible indicator value of the shape layer is usually higher, so the combinations of shapes and locations are used to encode. Meanwhile, the compressible indicator value of the detail layer is relatively lower, and other common coding methods can be adopted for encoding. The soft compression algorithm for a gray image consists of predictive coding, negative-to-positive mapping, layer separation, shape search, codebook generation and so on. We first introduce the overall architecture, followed by the vital steps.

#### 3.2.1. Overall Architecture

**Figure 2:**
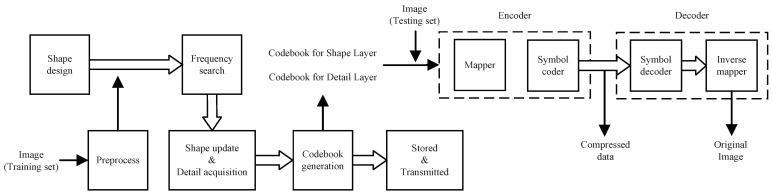
The overall procedure of soft compression algorithm for gray image including training and testing. The shape design, frequency search, shape update, detail acquisition, codebook generation, and saved codebook are the training stage. On the other hand, the encoder and decoder are the testing stage.

For soft compression algorithms, the codebook is very important. It directly determines the efficiency and performance of image compression. The algorithm consists of two parts, training and testing. The purpose of the training phase is to generate the codebook. In the testing phase, codebooks are used to encode and decode images to evaluate the performance of the algorithm. Figure 2 summarizes the overall architecture of the soft compression algorithm for a gray image. We design the set of shapes firstly, and then update the frequency of each candidate shape in the training set. After obtaining the final set of shapes and corresponding frequency, we generate the codebook for the shape layer. In training, we also acquire the codebook for the detail layer at the same time. Codebooks are used in the encoder and decoder, which are stored or transmitted for subsequent usage.

In testing, the image is compressed through the encoder. When the sender wants to communicate with the receiver, it firstly transmits the codebooks. After both sides of the communication have the same codebook, the transmitted content can be the compressed data instead of the original image. The receiver receives the compressed data after storage or transmission. Due to the completeness of codebooks, the recovered image is exactly the same as the original image, which ensures lossless compression.

#### 3.2.2. Predictive Coding and Negative-to-Positive Mapping

Predictive coding is an efficient way to transform spatial redundancy into coding redundancy with prediction. The core idea of predictive coding is to calculate the prediction value according to the spatial correlation, which aims to encode the prediction error. Negative-to-positive mapping maps the prediction error from negative to positive for layer separation.

**Figure 3:**
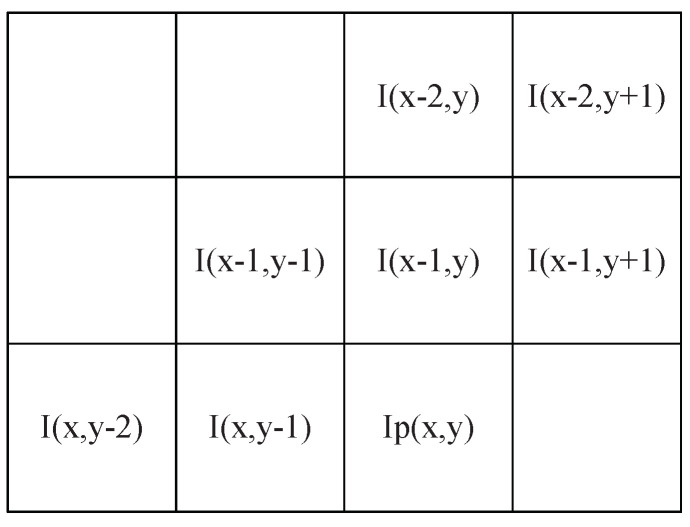
The spatial correlation between pixels with predictive coding.

Let I(x,y),$I_{P}\left(x,y\right)$ and $I_{E}\left(x,y\right)$ be the pixel intensity value, predictive value, prediction error at position (x,y) in an image *I*, respectively. We utilize the gradient adjusted prediction method [70] based on the gradient of the intensity function, which is shown in Formulas (42) and (43) (for simplicity, we use $I_{x,y}$ for I(x,y)). Figure 3 illustrates the spatial correlation between pixels.

After obtaining the predictive value, one can calculate the prediction error with Formula (44). The range of the prediction error is [−D+1,D−1], which is different from the pixel intensity value [0,D−1]. We use Formula (45) to map the prediction error from negative to positive, which is conducive to the subsequent procedures.

(42) $$\begin{array}{cc} \; & d_{h}=|I_{x,y-1}-I_{x,y-2}\left|+\right|I_{x-1,y}-I_{x-1,y-1}\left|+\right|I_{x-1,y}-I_{x-1,y+1}|\\ \; & d_{v}=|I_{x,y-1}-I_{x-1,y-1}\left|+\right|I_{x-1,y}-I_{x-2,y}\left|+\right|I_{x-1,y+1}-I_{x-2,y+1}|\\ \; & d_{s}=d_{v}-d_{h} \end{array}$$

(43) $$I_{P}\left(x,y\right)=\left\{\begin{array}{ccc} I_{x-1,y} & \; & \mathrm{if}\;d_{s}<-80\\ \frac{1}{4}I_{x,y-1}+\frac{3}{4}I_{x-1,y}+\frac{1}{8}I_{x-1,y+1}-\frac{1}{8}I_{x-1,y-1} & \; & \mathrm{if}\;-80\leq d_{s}<-32\\ \frac{3}{8}I_{x,y-1}+\frac{5}{8}I_{x-1,y}+\frac{3}{16}I_{x-1,y+1}-\frac{3}{16}I_{x-1,y-1} & \; & \mathrm{if}\;-32\leq d_{s}<-8\\ \frac{1}{2}I_{x,y-1}+\frac{1}{2}I_{x-1,y}+\frac{1}{4}I_{x-1,y+1}-\frac{1}{4}I_{x-1,y-1} & \; & \mathrm{if}\;-8\leq d_{s}\leq 8\\ \frac{5}{8}I_{x,y-1}+\frac{3}{8}I_{x-1,y}+\frac{3}{16}I_{x-1,y+1}-\frac{3}{16}I_{x-1,y-1} & \; & \mathrm{if}\;8<d_{s}\leq 32\\ \frac{3}{4}I_{x,y-1}+\frac{1}{4}I_{x-1,y}+\frac{1}{8}I_{x-1,y+1}-\frac{1}{8}I_{x-1,y-1} & \; & \mathrm{if}\;32<d_{s}\leq 80\\ I_{x,y-1} & \; & \mathrm{if}\;d_{s}>80 \end{array}\right.$$

(44) $$I_{E}\left(x,y\right)=I\left(x,y\right)-I_{P}\left(x,y\right)$$

(45) $$I^{\prime }\left(x,y\right)=\left\{\begin{array}{ccc} 2I_{E}\left(x,y\right) & \; & I_{E}\left(x,y\right)\geq 0\\ -2I_{E}\left(x,y\right)-1 & \; & I_{E}\left(x,y\right)<0 \end{array}\right.$$

The proportion of $r_{0}$ increases with predictive coding and mapping, which also leads to the increase in the compressible indicator value. The images will be more conducive to encoding with the soft compression algorithm. Another reason for adopting predictive coding and mapping is that the same image will have the same result after these two operations. The reversibility of each step ensures the losslessness of the soft compression algorithm.

#### 3.2.3. Layer Separation

Soft compression is suitable for images with a large compressible indicator value. After the previous steps, the compressible indicator value of an image is greatly improved. We continue to improve it. On the one hand, we observe that the probability of the prediction error decreases as the value increases. On the other hand, through a proper separation, one part with a higher compressible indicator value and another with a lower value can be generated.

Layer separation and bit-plane coding [71] are similar, but there are essential differences. Bit-plane coding focuses on decomposing a multilevel image into a series of binary images and compressing each binary image via one of several well-known binary compression methods. Bit-plane coding produces many layers. However, layer separation produces only two layers, shape layer $I_{S}^{\prime }$ and detail layer $I_{D}^{\prime }$.

$I^{\prime }$ is divided into shape layer $I_{S}^{\prime }$ and detail layer $I_{D}^{\prime }$ via Formulas (46) and (47).

(46) $$\begin{array}{c} I_{S}^{\prime }\left(x,y\right)=I^{\prime }\left(x,y\right)\;//\;2^{l} \end{array}$$

(47) $$\begin{array}{c} I_{D}^{\prime }\left(x,y\right)=I^{\prime }\left(x,y\right)\;\%\;2^{l} \end{array}$$

where // and % represent the quotient and remainder operation, respectively. Layer interface *l* is a constant between 0 and logD, which can be given in advance with searching or experience.

Because the compressible indicator value of the shape layer is usually higher, the combinations of locations and shapes are used for encoding. The compressible indicator value of the detail layer is relatively lower, and other coding methods, such as block coding and arithmetic coding, can be used for compression.

#### 3.2.4. Shape Search and Codebook Generation

The set of shapes with the soft compression algorithm directly determines the compression performance and coding efficiency of images. How to find the shape is vital. *A* is an m×n matrix whose components are in [0,2D−2]. $u_{i}$ and $v_{j}$ are vectors, representing the *i* row and *j* column of *A*, respectively. The matrix whose $u_{i}$ and $v_{j}$ follow (48) and (49) is suitable for designing shapes.

(48) $$\begin{array}{cc} \; & \left|\right|u_{i}{\left|\right|}_{0}\geq \frac{n}{2}\;\;\;\;\forall \;1\leq i\leq m \end{array}$$

(49) $$\begin{array}{cc} \; & \left|\right|v_{j}{\left|\right|}_{0}\geq \frac{m}{2}\;\;\;\;\forall \;1\leq j\leq n \end{array}$$

Formulas (48) and (49) indicate that the number of non-zero elements in all rows and columns must be no less than half of the row size and column size, respectively. By keeping only the non-zero value blocks in a matrix and removing the zero value blocks, one can obtain the candidate shape. This method can avoid the situation that different matrices generate the same shape.

**Figure 4:**
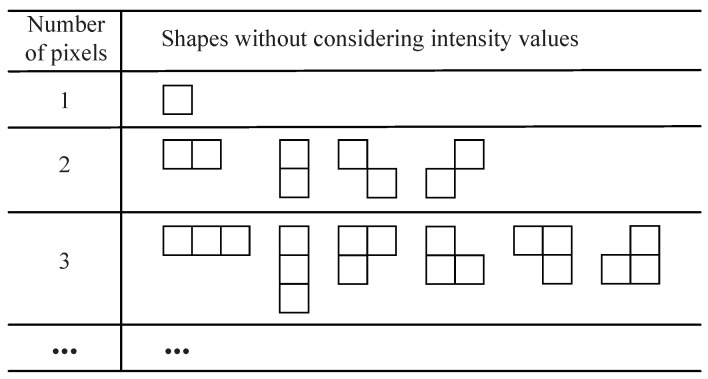
Shapes generated without considering intensity values according to the criteria (classified by the number of pixels). These are not all shapes in the set but only a part of it.

As illustrated in Figure 4, there is a part of shapes. These shapes are classified by size without considering intensity values. Combining them with the error intensity value [1,2D−2] can generate the shapes in actual use.

However, not all shapes appear in the final shape set. In fact, only a small number of shapes can be retained all the time. In training, the set of shapes is updated dynamically. The shape with less frequency is deleted in order to ensure that the size of shape set is controllable.

After obtaining the set of shapes, the codebook for the shape layer can be generated according to the frequency and size of each shape. While searching for the shape set, we also count the frequency distribution of the pixel intensity value in the detail layer so as to generate the codebook for the detail layer. The process of the training phase is visually shown in Algorithm 1, which connects the steps in Section 3.2.2, Section 3.2.3 and Section 3.2.4. When both sides of communication have the same codebook prior to the message exchanges, the transmitter can directly send compressed data instead of the whole image to the receiver, which is able to reduce the communication quantity and storage space. That is, it can save the communication bandwidth while keeping the same message change rate.
**Algorithm 1:** The training part of the soft compression algorithm for gray image.**Input:***W* images with size M×N**Output:** The codebook for the shape layer and detail layerPreprocess: predictive coding, negative-to-positive mapping and layer separation**for***Z*← 1 **to** *W* do **do**   **for** matrx size (u,v)←1×1**to**m×n, image coordinate (i,j)←1×1**to**M×N **do**     **if** $I_{S}^{\prime }\left[i,j:i+u,j+v\right]$ satisfies (48) **and** (49) **then**        Get the shape $S:=I_{S}^{\prime }\left[i,j:i+u,j+v\right]$        **if** *S* in the codebook **then**          Update the frequency of *S* in the codebook        **else**          Add *S* to the codebook        **end if**     **end if**   **end for**   Remove low-weight shapes based on frequency and size   Count the distribution of pixel values in the detail layer $I_{D}^{\prime }$**end for**Generate the codebooks

#### 3.2.5. Golomb Coding for Locations

A set of locations are generated when the shape layer is encoded. We use Golomb coding to encode the distance difference between each location and the previous location. Golomb coding [5] was designed for non-negative integer input with geometric probability distribution. We use it in the following steps.

- **Step 1.** Calculate the distance difference ∆ from the previous location.
- **Step 2.** Get a positive integer *m* by giving or searching in advance.
- **Step 3.** Form the unary code of quotient ⌊∆/m⌋. (The unary code of an integer *q* is defined as *q* 1s followed by a 0.)
- **Step 4.** Let $k=\lceil \log_{2}m\rceil$, $c=2^{k}-m$, r=∆modm, and compute truncated remainder $r^{\prime }$ such that
  (50) $$r^{\prime }=\left\{\begin{array}{ccc} r\;\mathrm{truncated}\;\mathrm{to}\;k-1\;\mathrm{bits}\;\; & \; & 0\leq r<c\\ r+c\;\mathrm{truncated}\;\mathrm{to}\;k\;\mathrm{bits}\;\; & \; & \mathrm{otherwise} \end{array}\right.$$
- **Step 5.** Concatenate the results of steps 3 and 4.

**Figure 5:**
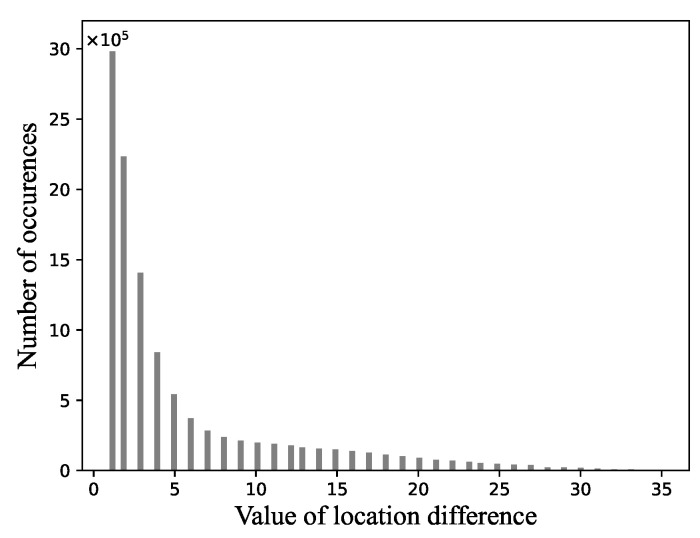
The frequency distribution of location difference on Fashion-MNIST dataset with soft compression algorithm for gray image.

The location difference obtained with the soft compression algorithm approximately obeys geometric probability distribution. Figure 5 shows the empirical frequency distribution on Fashion-MNIST dataset with the soft compression algorithm for gray image. Under the prior information, Golomb coding reduces the space for storing location differences.

#### 3.2.6. Encoder and Decoder

**Figure 6:**
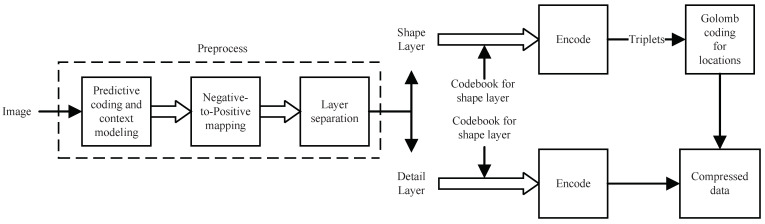
The procedure of encoder. It uses two different codebooks to encode the shape layer and the detail layer, respectively.

Encoder starts with preprocessing, which consists of the steps mentioned before (predictive coding, negative-to-positive mapping and layer separation). Secondly, two codebooks are used to encode the shape layer and detail layer, respectively. Thirdly, locations of the shape layer are encoded with Golomb coding. Finally, the compressed data can be obtained by connecting the coding results of the two layers. Figure 6 shows the entire process of the encoder.

**Figure 7:**
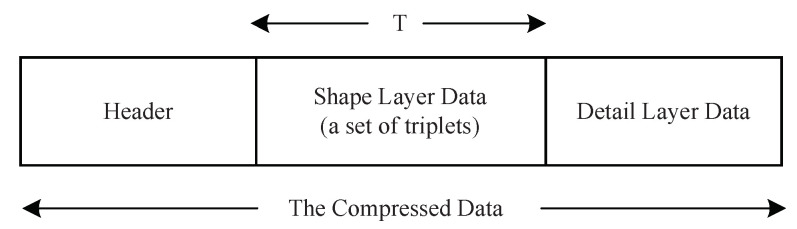
The composition of compressed data.

Figure 7 illustrates the composition of the compressed data. The header part contains information about the height and width of an image, as well as the layer interface. The shape layer data and detail layer data carry the encoding results of these two layers, respectively.

The decoder adopts the opposite structure to the encoder. The original image can be reconstructed through decoding. Firstly, the shape $S_{i}$ is obtained according to the codebook for the shape layer, and then the shape $S_{i}$ is filled in the location $\left(x_{i},y_{i}\right)$. The shape layer $I_{S}^{\prime }$ is acquired by repeating *T* operations. Secondly, the detail layer $I_{D}^{\prime }$ is decoded from the compressed data with the codebook for the detail layer. Finally, the original image can be reconstructed by merging $I_{S}^{\prime }$ and $I_{D}^{\prime }$, positive-to-negative mapping and anti-predictive coding.

#### 3.2.7. Concrete Example

**Figure 8:**
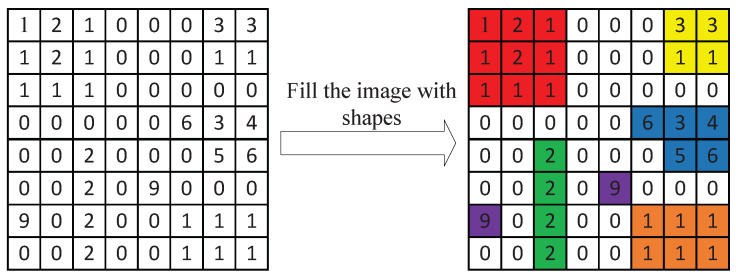
An example of the split of the shape layer. The image on the left is the original 8×8 shape layer. By filling the image with shapes in the codebook, it is divided into several shape regions. Each shape is marked with a different color, as shown on the right.

In order to describe the algorithm more intuitively, we use a specific example to illustrate the encoding process of the shape layer, in other words, how it changes from a digital image to the binary data. Figure 8 illustrates the process of filling an image with shapes in the codebook. The image on the left is an 8×8 shape layer. It is divided into several shapes. Each shape is marked with a different color, as shown on the right. We use the coordinate of the pixel in the upper left corner to represent the location of the whole shape. Therefore, the locations of these shapes are $\left\{\left(x_{i},y_{i}\right):\left(0,0\right),\left(0,6\right),\left(3,5\right),\left(4,2\right),\left(5,4\right),\left(6,0\right),\left(6,5\right)\right\}$. Using relative values to represent locations can reduce coding costs. In this case, the locations can be re-expressed as $\left\{\left(x_{i},y_{i}\right):\left(0,0\right),6,23,5,10,4,5\right\}$. Except for the first location, the rest are represented by relative values (difference from the previous location).

**Figure 9:**
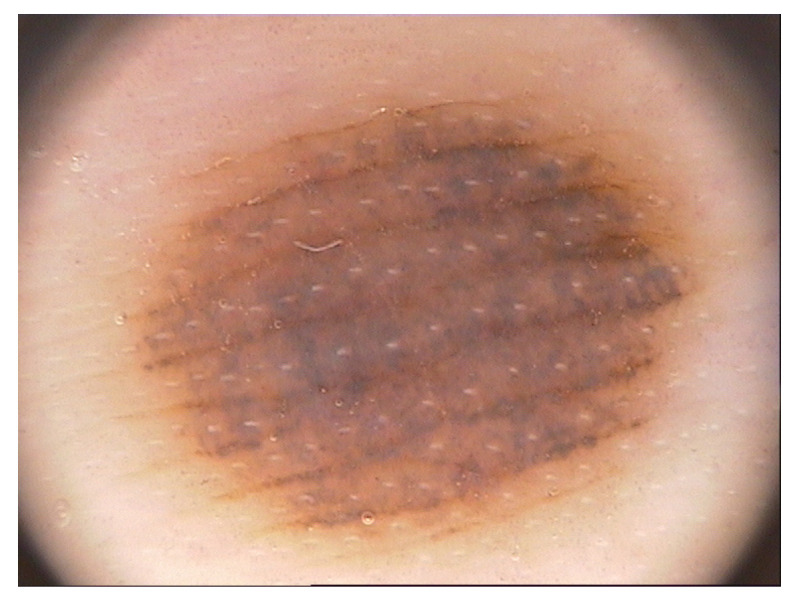
An example of image encoding with soft compression, PNG, JPEG2000 and JPEG-LS. It is a dermoscopic image from medical datasets. The compression ratios are 2.53, 1.85, 2.41 and 2.45, respectively.

After the image is divided into shapes and their corresponding locations, it is necessary to convert them into binary data. We use natural binary coding for the first location and Golomb coding (Section 3.2.5) for the rest locations. Therefore, the binary data for each location are $\left\{{\left(x_{i},y_{i}\right)}_{bit}:000000,1010,11111011,1001,11010,1000,1001|m=4\right\}$. In training, we assign a one-to-one corresponding codeword to each shape in the codebook. There are seven shapes in Figure 8, two of which are the same. We need to find the codeword corresponding to each shape from the codebook, which is the binary data representation. For example, the result of combining each codeword and location may be $\{\left({\left(x_{i},y_{i}\right)}_{bit},C_{i}\right):\left(000000,000\right),\left(1010,001\right),\left(11111011,010\right),$(1001,011),(11010,100),(1000,100),(1001,101)}. The second item in each bracket represents the codeword corresponding to the shape. Connecting all the small binary fragments is the binary data of the shape layer. On the other hand, for clearly showing the compression ratio improvement, we use a complete image encoding example. Figure 9 illustrates its compression ratios with soft compression, PNG, JPEG2000 and JPEG-LS. The compression ratios are 2.53, 1.85, 2.41 and 2.45, respectively. This example indicates the improvement in the compression ratio of soft compression.

### 3.3. Multi-Component Image

Considering a multi-component image, the soft compression algorithm for a gray image can be used for each component. In this case, the soft compression algorithm for a multi-component image is equivalent to the combination of several gray images. In addition, the compressed data are also a combination of several components.

## 4. Experimental Results and Theoretical Analysis

In this section, we reveal the experimental results and theoretical analysis of the soft compression algorithm for a binary image, gray image and multi-component image.

**Definition** **5.** *Suppose that b and $b^{\prime }$ represent the required number of bits to store the same image with natural binary code and other coding methods, respectively. The compression ratio R is defined as*

(51) $$R=\frac{b}{b^{\prime }}$$

*and $R_{avg}$ is defined as the average compression ratio of a class of images.*

The average compression ratio $R_{avg}$ reflects the performance of different encoding methods. We adopt it as an significant criterion to measure the image compression algorithm.

### 4.1. Binary Image

We tested the soft compression algorithm for a binary image [1] on the MNIST [72] dataset. As expected, it had excellent results. In this subsection, we analyze the experimental results theoretically. The MNIST dataset has 10 classes. Each category of images may have a different compressible indicator value. Although they are of different classes, the compressible indicator value is generally subject to the normal distribution.

Table 1 illustrates the experimental results of the MNIST dataset with the soft compression algorithm for a binary image (each class uses the same codebook). CIV and $R_{avg}$ are strongly related, and their Pearson correlation coefficient is 0.977. The larger the average compressible indicator value, the greater the average compression ratio. The results are consistent with the theoretical analysis in Theorem 2. It enlightens us to the fact that soft compression is suitable for compressing images with a large compressible indicator value. Although the compression ratio is not only determined by this factor, the compressible indicator value is the key element affecting the compression performance.

### 4.2. Gray Image and Multi-Component Image

In this subsection, we obtain the experimental results on different datasets with the soft compression algorithm. The compression ratio is one of the most important criteria to evaluate the performance of the encoding algorithm. Table 2 illustrates the average compression ratio of each class in Fashion-MNIST [73] with different methods. We emphasize the difference in percentage to soft compression for each other method in green if soft compression outperforms the other method. Our algorithm outperforms the widely-used PNG on all classes, which is a 24% improvement, on average. It also outperforms JPEG2000 and JPEG-LS, with 48% and 6.8% improvements, respectively.

Moreover, Table 3 shows the experimental results on datasets with larger images. The DRIVE [74] dataset is obtained from a diabetic retinopathy screening program to study skin lesions. PH2 [75] is a dermoscopic image database. PNG, JPEG2000 and JPEG-LS are the most popular methods in lossless image compression. L3C [46] is a novel learned lossless image compression system based on deep neural networks. We compare soft compression with these algorithms. In terms of the average compression ratio, soft compression outperforms any other method. On the other hand, soft compression and other methods have their own advantages for the maximum and minimum. The results of Table 2 and Table 3 indicate that the soft compression algorithm is better than some known classical methods, PNG, JPEG2000, JPEG-LS and L3C, in terms of the compression ratio. For the same kind of image, it is better to choose soft compression to encode.

Table 4 illustrates the average compression ratio of the soft compression algorithm for a gray image on Fashion-MNIST. It is the result of cross validation. The first column denotes codebooks, which are generated by the training set of each class separately. The first row represents each category of the testing set. The value on (i,j) denotes the average compression ratio $R_{avg}$ of the *j*-th class of the testing set by using the codebook generated by the *i*-th class of the training set. For example, the value on (Trouser, T-shirt) is the average compression ratio of T-shirt’s testing set, whose codebook is trained on Trouser’s training set. It is observed that the values on the diagonal are higher than those of the same column, which suggests that soft compression is related to the matching degree between the dataset and the codeook. The higher the matching degree, the higher the compression ratio.

In practice, it is better to adopt a corresponding codebook for the specific category of images. However, imperfect matching may reduce the compression ratio, but will not cause any loss of information. In fact, codebooks of soft compression are complete. That is to say that for any codebook and any picture, lossless compression can always be achieved. The difference lies in diverse compression ratios. From Table 4, we can draw a conclusion that the compression ratio is both related to images and codebooks. This corresponds to the compressible indicator value and the similarity between images and codebooks. The larger the compressible indicator value, the higher the similarity between images and codebooks, and the higher the compression ratio.

**Figure 10:**
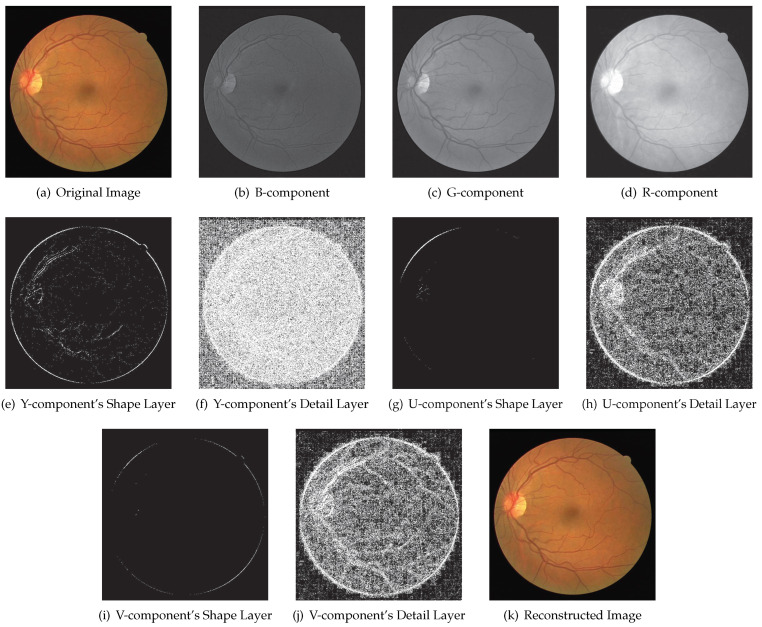
An example of DRIVE dataset with soft compression algorithm for multi-component image. (**a**) The original RGB image. (**b**) B-component image obtained by separation. (**c**) G-component image obtained by separation. (**d**) R-component image obtained by separation. (**e**) The shape layer (binarization) of Y-component. (**f**) The detail layer (binarization) of Y-component. (**g**) The shape layer (binarization) of U-component. (**h**) The detail layer (binarization) of U-component. (**i**) The shape layer (binarization) of V-component. (**j**) The detail layer (binarization) of V-component. (**k**) The reconstructed image.

For a multi-component image, its process is the combination of several gray images. Figure 10 illustrates an example with the soft compression algorithm for a multi-component image. Subfigure (a) is a multi-component original image, whose components are B, G and R, respectively, as shown in (b), (c) and (d). After dividing each component into the shape layer and detail layer, respectively (binarization was made for a clearer appearance), one can obtain subfigures (e) to (j). Furthermore, the compressed file can be generated by adopting the above-mentioned encoding method for the shape layer and detail layer. The reconstructed subfigure (k) can be obtained through decoding, which is the same as the original subfigure (a).

### 4.3. Implementation Details

The architecture of the soft compression algorithm is shown in Figure 2. Detailed implementation can be found in the code. The algorithm is implemented with Python on a single Intel i7-9700K CPU. The batch size is 10 for Fashion-MNIST and 1 for the other two datasets. In addition, the shape degree is set to 0.1 for Fashion-MNIST and 0.5 for the other three datasets.

The encoding and decoding complexity are both related to the image size and the number of shapes in the codebook, i.e., O(MN|S|). Moreover, the average encoding and decoding times of an image are shown in Table 5.

## 5. Conclusions

In this paper, we investigated how to apply soft compression to encode images in the lossless mode. It uses shapes to encode an image, which aims to eliminate coding redundancy and spatial redundancy at the same time. Due to the adaptability and completeness of codebooks with soft compression, it can always achieve lossless compression for any image and any codebook.

In theory, we also proposed a new concept, the compressible indicator function with regard to images, and theoretically illustrated the working principle. The compressible indicator function points out the suitable scenarios of soft compression. In addition, it also gives a threshold about the required number of bits to represent a location with soft compression.

Moreover, we designed soft compression algorithms for a binary image, gray image and multi-component image. These algorithms were tested on the datasets. Experimental results indicated that soft compression has significant effects on lossless image compression, which outperform classical systems PNG and JPEG2000, especially for images which have a large compressible indicator value.

This paper focuses on lossless compression. However, soft compression can also be combined with other transformation methods, such as wavelet transform. Lossy compression can be realized by using soft compression for the coefficients of transform domain. Soft compression can also be combined with channel coding to enhance the effect of joint source-channel coding.

It is expected that this work may have excellent applications when errors cannot be tolerated or where there is critical social or scientific value, such as CT image processing for the diagnosis and treatment of medical image files, digital libraries and so on.

## Figures and Tables

**Table 1:** Average compressible indicator value and compression ratio of MNIST dataset with soft compression algorithm for binary image (each class uses the same codebook).

Class	0	1	2	3	4	5	6	7	8	9
CIV	3.87	5.14	4.09	4.17	4.42	4.30	4.17	4.51	4.06	4.37
Compression ratio	**2.84**	**6.02**	**3.17**	**3.20**	**3.77**	**3.40**	**3.20**	**4.05**	**2.81**	**3.52**

**Table 2:** Average compression ratio of Fashion-MNIST with different image compression methods (images are gray and all methods are lossless compression).

Class	Method			
	Soft Compression	PNG	JPEG2000	JPEG-LS
T-Shirt	**1.53**	1.23 +24%	1.06 +44%	1.47 +4.1%
Trouser	**2.30**	1.50 +53%	1.32 +74%	2.13 +8.0%
Pullover	**1.48**	1.12 +32%	1.02 +45%	1.36 +8.8%
Dress	**1.85**	1.41 +31%	1.20 +54%	1.79 +3.4%
Coat	**1.45**	1.14 +27%	1.03 +41%	1.36 +6.7%
Sandals	**1.95**	1.82 +7.1%	1.33 +47%	1.82 +7.1%
Shirt	**1.42**	1.14 +25%	1.03 +38%	1.34 +6.0%
Sneaker	**2.07**	1.88 +10%	1.39 +49%	1.89 +9.5%
Bag	**1.50**	1.32 +14%	1.07 +40%	1.42 +5.6%
Ankle boots	**1.66**	1.46 +14%	1.14 +46%	1.52 +9.2%

**Table 3:** The compression results of DRIVE and PH2 datasets with different image compression methods (all methods are lossless compression). DRIVE is divided into training set and testing set according to the original division method. PH2 was not divided before, so the training set and testing set are divided in a 5:3 manner. L3C selects the best performing model among the provided models.

Dataset	Statistic	Method				
		Soft Compression	PNG	JPEG2000	JPEG-LS	L3C
DRIVE [74] 565 × 584 px	Mean	**3.201**	2.434 +32%	2.972 +7.7%	3.064 +4.5%	2.989 +7.1%
	Minimum	**2.893**	2.331 +24%	2.790 +3.7%	2.731 +5.9%	2.841 +1.8%
	Maximum	**4.171**	2.760 +51%	3.671 +14%	3.941 +5.8%	3.604 +16%
	Variance	0.0657	0.0072	0.0333	0.0632	0.0287
PH2 [75] 767 × 576 px	Mean	**2.570**	1.727 +49%	2.450 +4.9%	2.488 +3.3%	2.300 +12%
	Minimum	1.686	1.501	**1.812**	1.737	1.790
	Maximum	**3.388**	2.021 +68%	2.975 +14%	3.045 +11%	2.920 +16%
	Variance	0.1538	0.0108	0.0749	0.0835	0.1047

**Table 4:** Average compression ratio of Fashion-MNIST dataset with soft compression algorithm for gray image (each class has its own codebook).

Class	T-Shirt	Trouser	Pullover	Dress	Coat	Sandals	Shirt	Sneaker	Bag	Ankle Boots
T-shirt	1.55	2.19	1.50	1.83	1.48	1.90	1.44	2.00	1.51	1.65
Trouser	1.48	2.35	1.43	1.82	1.41	1.91	1.38	2.03	1.46	1.61
Pullover	1.55	2.20	1.50	1.82	1.48	1.88	1.44	1.99	1.51	1.65
Dress	1.54	2.32	1.48	1.87	1.46	1.96	1.43	2.08	1.51	1.66
Coat	1.54	2.20	1.50	1.83	1.48	1.88	1.44	2.00	1.51	1.65
Sandals	1.53	2.27	1.47	1.85	1.45	2.01	1.42	2.11	1.51	1.68
Shirt	1.55	2.20	1.50	1.83	1.48	1.89	1.44	2.00	1.51	1.65
Sneaker	1.52	2.27	1.46	1.84	1.45	1.99	1.41	2.11	1.51	1.67
Bag	1.55	2.25	1.49	1.84	1.47	1.94	1.44	2.06	1.53	1.67
Ankle boots	1.54	2.24	1.49	1.84	1.47	1.94	1.43	2.06	1.51	1.67

**Table 5:** Average encoding and decoding times of images with soft compression algorithm.

	Fashion-MNIST [73] 28 × 28 px	DRIVE [74] 565 × 584 px	PH2 [75] 767 × 576 px
Encoding	5.7 × $10^{-2}$ s	7.79 s	24.98 s
Decoding	4.1 × $10^{-3}$ s	5.31 s	6.80 s

## Data Availability

The code of soft compression for gray image is available: https://github.com/ten22one/Soft-compression-algorithm-for-gray-image (accessed on 9 December 2021).
